# Inferior Vestibular Neuritis: Diagnostic Criteria, Clinical Features, and Prognosis—A Focused Review

**DOI:** 10.3390/medicina61020361

**Published:** 2025-02-19

**Authors:** Gabriela Cornelia Musat, Mihai Alexandru Preda, Ionut Tanase, Adina Zamfir Chiru Anton, George G. Mitroi, Ovidiu Musat, Alina Lavinia Antoaneta Oancea, Mihaela Roxana Mitroi

**Affiliations:** 1 Department of Otorhinolaryngology, Carol Davila University of Medicine and Pharmacy, 020021 Bucharest, Romania; gabriela.musat@umfcd.ro (G.C.M.); mihai.preda@umfcd.ro (M.A.P.); ionut.tanase@umfcd.ro (I.T.); zamfiradina@yahoo.com (A.Z.C.A.); dr.alina.oancea@gmail.com (A.L.A.O.); 2 Department of Dermatology, Faculty of Medicine, University of Medicine and Pharmacy of Craiova, 200349 Craiova, Romania; mitroi.george@yahoo.com (G.G.M.); mihaela.mitroi@umfcv.ro (M.R.M.); 3 Department of Ophtalmology, Carol Davila University of Medicine and Pharmacy, 020021 Bucharest, Romania

**Keywords:** vestibular neuritis, inferior vestibular neuritis, vHIT, VEMP, caloric testing

## Abstract

*Purpose*: This review aims to analyze the diagnostic methods used to evaluate inferior vestibular nerve neuritis. *Methods*: We performed an electronic search on the PubMed database for clinical studies investigating the diagnostic techniques used for inferior vestibular nerve neuritis. *Results*: We initially identified 114 records in our search. After applying the inclusion and exclusion criteria, we narrowed it down to 12 studies. These studies collectively examined a total of 642 patients diagnosed with vestibular nerve neuritis, 64 of whom had inferior vestibular neuritis. *Conclusions*: The inferior vestibular neuritis is an unfrequent form of vestibular neuritis, often misdiagnosed. The diagnosis of inferior vestibular neuritis (IVN) is based on clinical history and vestibular testing, specifically reduced posterior canal gain on video head impulse test (vHIT), absent cervical VEMP (cVEMP), normal caloric responses, and preserved ocular VEMP (oVEMP). This review highlights the gaps in current diagnostic strategies and emphasizes the need for integrating advanced vestibular testing methods to enhance diagnostic accuracy for inferior vestibular nerve neuritis. Future studies should address the standardization of diagnostic protocols to facilitate broader clinical application.

## 1. Introduction

Inferior vestibular neuritis (IVN) is an underdiagnosed form of vestibular neuritis characterized by selective dysfunction of the inferior division of the vestibular nerve. Unlike superior vestibular neuritis (SVN), which affects the horizontal and anterior semicircular canals, IVN involves the posterior semicircular canal and saccule, leading to a distinct clinical presentation. This review aims to clarify the diagnostic criteria, clinical findings, and differentiation of IVN from SVN and central disorders. As the pathophysiology was not elucidated, different authors suggested other terms such as “vestibular neuropathy”, “unilateral sudden partial loss of vestibular function”, “acute vestibulopathy”, “vestibular failure”, “acute unilateral vestibulopathy”, and “acute peripheral vestibulopathy”. The Barany Committee for the Classification of Vestibular Disorders prefers the term “acute unilateral vestibulopathy” but the term “vestibular neuritis” can be used simultaneously because it is already widely used [1]. Although the etiology is not completely elucidated, at least for some cases, it is considered to be the result of a reactivation of a herpes simplex infection [2]. The vascular or autoimmune etiology cannot be excluded with certainty [3].

Vestibular neuritis has a reported incidence ranging between 3.5 and 15.5 per 100,000 population [4,5]. Although it was previously thought that women are more affected than men, current evidence suggests that both sexes are affected in equal proportion [6,7]. The onset of the disease is typically reported between 30 and 60 years, with a plateau occurring between 40 and 60 years [5,6].

The diagnosis of vestibular neuritis is based on correlating clinical signs with laboratory examinations, as there is no definitive confirmatory test available. Since only one division of the vestibular nerve may be affected, three distinct clinical presentations can occur: total vestibular neuritis, superior vestibular neuritis, and inferior vestibular neuritis. Symptoms such as vertigo and imbalance are non-specific, and signs like nystagmus can be difficult to evaluate, leading to potential misdiagnosis. Laboratory tests used to assess vestibular function in patients with vestibular neuritis include VNG with caloric or rotational testing, vHIT, and VEMP.

It is crucial to correctly distinguish between central and peripheral vestibular disorders, as treatment depends on this distinction. Central disorders often require immediate intervention, whereas peripheral conditions, such as vestibular neuritis, are usually managed conservatively. Misdiagnosis can lead to treatment delays, increased healthcare costs, and unnecessary patient distress. Inferior vestibular neuritis is frequently mistaken for a central vestibular disorder.

Unfortunately, research on inferior vestibular neuritis is limited, making it difficult to establish a clear and unified understanding of the condition. The aim of our systematic review is to consolidate fragmented information, summarize current diagnostic methods, identify existing knowledge gaps, and, ultimately, enhance the diagnosis and management of this condition.

Future research should focus on the specific challenges in diagnosing inferior vestibular neuritis, particularly in distinguishing it from superior vestibular neuritis and central vestibular disorders. This differentiation is critical for improving patient outcomes and reducing misdiagnosis rates.

## 2. Materials and Methods

We conducted a comprehensive search in the PubMed electronic database, with no time constraints, concluding on 2 September 2024. The search strategy used was ((vestibular neuritis) AND (inferior vestibular neuritis)) AND (diagnosis). Titles were screened based on the inclusion and exclusion criteria outlined below. While guidelines and systematic reviews were reviewed for background context, only primary research studies providing new diagnostic insights on IVN were included in the final analysis.

The inclusion criteria were:

- Population: Adults (18+ years) diagnosed with or suspected of having inferior vestibular nerve neuritis or presenting with symptoms suggestive of this condition.
- Concept: Focus on diagnosis, misdiagnosis, clinical features, and differentiation between inferior vestibular nerve neuritis (peripheral) and central vestibular disorders.
- Context: Studies exploring diagnostic tools (e.g., clinical examination, vestibular testing, imaging) and cases of misdiagnosis between peripheral and central disorders.
- Types of Studies: All study designs, including case reports, cohort studies, diagnostic accuracy studies, reviews, and guidelines.

The exclusion criteria were as follows:

- Language and Abstract: Only papers written in English were taken into consideration. Articles without abstracts were excluded.
- Studies focused exclusively on superior vestibular neuritis.
- Pediatric populations or studies with participants under 18 years of age.
- Articles that did not discuss the diagnostic process or differentiation between inferior vestibular nerve neuritis and central disorders.
- Studies using a non-validated diagnostic approach.

## 3. Results

### 3.1. Overall Results

The search process initially identified 214 titles. After excluding non-English articles, 196 unique titles remained for evaluation. Each publication was screened based on its title and abstract to assess its relevance to the topic. A total of 114 titles were excluded for being unrelated or insignificant to our study.

Full-text access was obtained for 82 articles identified as potentially relevant. Each article was carefully reviewed to determine whether it met the inclusion criteria for the quantitative analysis. After a thorough assessment, 12 articles met the requirements and were included in the final review.

To ensure the rigor of the screening process, two independent reviewers assessed the articles. In cases of disagreement, a third reviewer was consulted to resolve differences and maintain the reliability of the selection process.

However, a potential limitation of this review should be acknowledged. The findings may be influenced by biases in the selected studies, including differences in study design, sample sizes, or diagnostic criteria, which could affect the generalizability of the results.

Our quantitative analysis is based on 12 studies that explore key research on vestibular neuritis and highlight the role of various diagnostic tools, including vestibular evoked myogenic potentials (VEMP), video head impulse testing (vHIT), and caloric testing.

**Figure 1:**
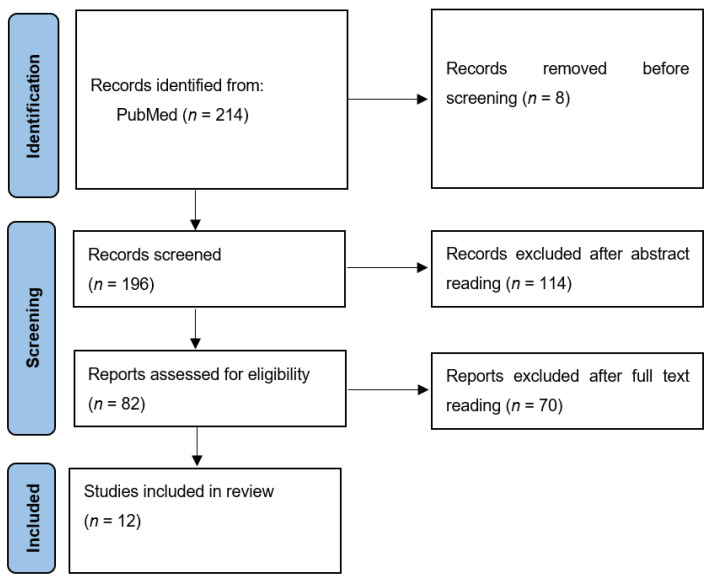
Flow chart of the literature search.

The process of selection of the articles is illustrated in the flow diagram in Figure 1.

### 3.2. Early Insights into Vestibular Neuritis

Aw et al. [8] conducted an analysis of 33 patients, identifying total vestibular neuritis in 21 cases, superior division dysfunction in 8 patients, and inferior division dysfunction in only 2 patients. Eight patients exhibited deficits in all three semicircular canals, indicating involvement of both the superior and inferior vestibular nerves. In 21 patients, deficits were observed in the lateral semicircular canal or a combination of the lateral and anterior canals, consistent with selective involvement of the superior vestibular nerve. Meanwhile, two patients who also had hearing loss on the same side showed normal caloric test results but exhibited isolated posterior semicircular canal deficits on vHIT. The authors suggested that these cases represent selective dysfunction of the inferior vestibular nerve. The study demonstrated that vHIT is a valuable diagnostic tool, as it can detect dysfunction in the vertical semicircular canals, thereby enabling the diagnosis of inferior vestibular neuritis [8].

Halmagyi et al. [9] described two cases of isolated inferior VN, marking an important step in distinguishing between the two subtypes. The authors presented two cases of patients with acute vertigo who exhibited normal lateral semicircular canal function, confirmed by both impulsive and caloric testing. However, both showed selective dysfunction of the posterior semicircular canal, as indicated by impulsive testing, and saccular dysfunction, as shown by vestibular evoked myogenic potential testing. These findings suggested a diagnosis of selective inferior vestibular neuritis. These cases supported the notion that unilateral lateral semicircular canal dysfunction is not a prerequisite for diagnosing acute vestibular neuritis in patients with acute spontaneous vertigo. By combining VEMP, vHIT, and caloric testing, the authors established a diagnostic framework for identifying inferior division involvement, which had previously been challenging to diagnose [9].

Monstad et al. [10] further highlighted the diagnostic challenges associated with inferior VN. They described three patients presenting with symptoms consistent with vestibular neuritis but normal caloric test results. All three presented unilateral loss of VEMP responses and a slight asymptomatic position-dependent nystagmus, occurring when the affected ear was positioned downward. In conclusion, the findings were interpreted as suggesting that these patients had isolated inferior vestibular nerve neuritis [10].

### 3.3. Larger Cohort Studies

Zhang et al. [11] conducted a significant study of 216 patients, identifying 8 cases of inferior vestibular neuritis based on a detailed analysis of diagnostic test results. These eight patients exhibited clinical features consistent with vestibular neuritis. Pure tone audiometry and caloric testing results were normal, and central lesions were ruled out through cerebral CT or MRI performed at admission. Six of these patients demonstrated unilateral loss of cervical vestibular evoked myogenic potentials, while two showed a unilateral reduction in VEMP amplitude. This study highlights the utility of combining VEMP and caloric testing to differentiate between superior and inferior vestibular neuritis [11].

Similarly, Nola et al. [12] investigated twenty patients with acute vertigo due to vestibular neuritis. Assessments, including Dix–Hallpike and Pagnini–McClure maneuvers, head-shaking tests, pure-tone audiometry, tympanometry, caloric testing (Fitzgerald–Hallpike method), and VEMP, were performed during the acute attack and at 8 days, 1 month, and 3 months post-onset. At the final follow-up, the 11 patients diagnosed with superior branch vestibular neuritis exhibited no improvement in caloric responses but maintained normal VEMP amplitude and latency bilaterally. In contrast, the 9 patients with inferior branch vestibular neuritis showed VEMP reflex recovery and normal caloric test results. The study highlights the value of VEMP as a practical screening tool for VN diagnosis, providing critical information, especially when caloric test results are inconclusive [12].

Chihara et al. [13] examined a retrospective case series, analyzing caloric test and cVEMP responses in 71 patients diagnosed with vestibular neuritis. Patients were categorized into three groups: the inferior vestibular neuritis group with asymmetrical cVEMP responses (13 patients), the superior vestibular neuritis group with asymmetrical caloric responses (34 patients), and the total vestibular neuritis group with asymmetrical responses in both tests. The study emphasizes that inferior vestibular neuritis is a relatively minor subtype of vestibular neuritis, characterized by downbeating torsional nystagmus beating toward the healthy ear, selective posterior semicircular canal dysfunction on vHIT, and absent cVEMP responses indicating saccular involvement [13].

### 3.4. Refining Diagnostic Techniques

Shin et al. [14] explored the role of both cervical and ocular VEMPs in 41 patients with acute vestibular neuritis. In this study, all 30 patients diagnosed with superior vestibular neuritis exhibited normal cervical vestibular evoked myogenic potentials (cVEMPs), indicating that the saccular receptors and their afferents in the inferior vestibular nerve were preserved. However, all these patients showed abnormal ocular vestibular evoked myogenic potentials (oVEMPs). In contrast, patients with inferior VN displayed normal oVEMP results but abnormal cVEMPs, suggesting selective dysfunction of the inferior vestibular nerve affecting the saccular receptors. The study highlighted the complementary roles of oVEMP and cVEMP in assessing the utricle and saccule, respectively [14].

Xie et al. [15] provided a unique case study of inferior vestibular neuritis in a fighter pilot. Despite the rarity of the condition, vHIT proved instrumental in confirming the diagnosis, showcasing its utility even in isolated cases [15].

Taylor et al. [16] examined 43 patients and identified superior vestibular neuritis in 41.9% of cases, total vestibular neuritis in 55,8%, and inferior vestibular neuritis in 2.3% (1 patient). This study emphasized the importance of combining VEMP and vHIT for comprehensive evaluation, as each test provided unique insights into vestibular function [16].

### 3.5. Recent Developments and Comprehensive Testing

Büki et al. [17] focused on 44 patients with vestibular neuritis, finding 19 cases of superior vestibular neuritis and 4 cases of inferior vestibular neuritis. The study shed light on how semicircular canal involvement can influence recovery trajectories, offering valuable insights into patient prognosis. A secondary aim of the study was to assess the utility of the HINTS test in the differential diagnosis of vestibular failure. In cases of inferior vestibular neuritis and isolated cases of horizontal canal involvement, the HINTS test may be false positive, indicating a cerebellar stroke [17].

Lee et al. [18] conducted a large-scale study involving 133 patients, identifying 64 cases of superior VN and 16 of inferior VN. The study reinforced the diagnostic value of combining caloric, vHIT, and VEMP testing for accurate localization of vestibular dysfunction. Using the vHIT in diagnosing acute vestibular neuritis can facilitate the identification of the rare subtype of inferior vestibular neuritis. This is especially important because inferior vestibular neuritis can sometimes resemble central nervous system disorders, which might lead to misdiagnosis [18].

Yacovino et al. [19] provided a modern perspective on vestibular neuritis in a cohort of 35 patients. They identified superior vestibular neuritis in 20 cases and total vestibular neuritis (involving both superior and inferior divisions) in 10 cases. Their findings show the importance of integrating advanced vestibular testing techniques to achieve a more thorough understanding of the condition. Using vHIT and VEMP testing to examine all 10 vestibular end organs is considered by the authors the fastest and most effective way to identify lesion patterns. This approach allows for precise differentiation between unilateral, bilateral, superior division, inferior division, or single-branch involvement. By providing a more detailed understanding of the lesion pattern, it enables a more accurate diagnosis of acute vestibular neuritis [19].

We summarize the information presented above in Table 1.

The review discusses 12 studies focused on patients with vestibular neuritis, involving 642 individuals. Among them, 64 patients (9.96%) were diagnosed with inferior vestibular neuritis. Most studies (9 out of 12) used VEMP testing to identify dysfunction in the inferior branch of the vestibular nerve, while 7 studies relied on vHIT findings for diagnosis. In some cases, the HINTS test can be misleading in IVN. While nystagmus and skew deviation components may suggest a peripheral cause, the normal horizontal HIT can create diagnostic uncertainty, as a positive HIT is typically expected in vestibular neuritis. This may falsely suggest a central lesion, particularly if downbeat nystagmus is also present.

## 4. Discussion

### 4.1. Cross-Analysis

The anatomy of the inner ear and vestibular nerve provides insight into the patterns of vestibular nerve involvement during inflammatory processes. The superior division of the vestibular nerve gathers input from the superior semicircular canal, horizontal semicircular canal, and utricle, while the inferior division transmits signals from the posterior semicircular canal and saccule. There is ongoing debate about whether the saccule is exclusively innervated by the inferior vestibular nerve or if it also receives partial input from the superior vestibular nerve, which may explain variability in cVEMP responses.

Goebel et al. [20] showed in a study in 2001 that the bony canal of the superior vestibular nerve is longer than that of the singular nerve (a branch of the inferior vestibular nerve). Furthermore, the superior vestibular nerve and its accompanying arteriole pass through a narrower pathway compared to the singular nerve and its vascular supply. Anatomically, this makes the superior division of the vestibular nerve more vulnerable to entrapment and potential ischemic changes within the labyrinth, thus explaining why superior vestibular neuritis is more frequent compared to inferior vestibular neuritis [20].

According to Gianoli’s 2005 study [21], the bony channel of the superior division of the vestibular nerve is seven times longer than that of the inferior division. Additionally, the superior nerve division contains more spicules inside the bony canal, resulting in a higher percentage of the channel occupied by bone, especially at the midpoint. This longer bony channel, combined with the increased presence of spicules, makes the superior nerve more exposed to entrapment and ischemia [21].

In 2021, Büki et al. [22] published a study implying that while the difference in channel length might play a role in why vestibular nerves are differently affected, there are also other factors that may explain why the superior vestibular nerve is more vulnerable. The superior vestibular nerve is more vulnerable due to its longer bony canal, narrower passage, and greater susceptibility to ischemic damage. In contrast, the inferior vestibular nerve appears to be less vulnerable, possibly due to the overlapping innervation of the saccule and posterior semicircular canal, which may provide some functional redundancy and resilience [22].

Based on the affected branch of the vestibular nerve, three types of clinical presentations can be observed. Total vestibular neuritis occurs when the entire nerve is affected, while superior and inferior vestibular neuritis are variants in which only one division of the nerve is involved.

The clinical presentation of vestibular neuritis varies depending on which division of the vestibular nerve is affected. The most common type is superior vestibular neuritis [23,24], while inferior vestibular neuritis is rare. Unlike superior vestibular neuritis, inferior vestibular neuritis lacks some of the typical findings seen in the more common form, making it more likely to be mistaken for a central vestibular disorder [25]. Patients with vestibular neuritis typically present with acute vertigo, instability, and nausea/vomiting. In some cases, they report a preceding or concurrent viral illness.

Usually, when the whole nerve is affected, individuals with vestibular neuritis exhibit a mixed horizontal–torsional nystagmus beating away from the affected ear, which is observed during the physical examination. The torsional component of nystagmus can be subtle and difficult to observe, making it appear predominantly horizontal, similar to what is seen in conditions affecting only the horizontal canal or its afferent pathways [26]. This nystagmus must be carefully differentiated from pseudo-spontaneous nystagmus, a type of nystagmus that might appear in the lateral canal paroxysmal positional nystagmus ageotropic variant. Pseudo-spontaneous nystagmus is a horizontal–torsional nystagmus that changes with head position. It increases when turning toward the affected ear and decreases when turning toward the healthy ear. In IVN, it is downbeat–torsional, while in SVN, it is horizontal–torsional [19]. Vestibular neuritis nystagmus is inhibited by visual fixation, has a fixed direction, and increases when the patient looks to the healthy side. Thus, it exhibits the characteristics of peripheral vestibular nystagmus. The nystagmus in vestibular neuritis is intensified by a head-shaking test, vibration, or hyperventilation [27,28,29]. The patient tends to fall toward the diseased side when standing or when attempting to walk. However, in IVN, the most reliable pathological findings are abnormal vHIT for the posterior semicircular canal, absent cVEMP (saccular dysfunction), and normal caloric responses. Head-shaking nystagmus is not essential for IVN diagnosis, as spontaneous nystagmus is already present in the acute phase.

Spontaneous horizontal–vertical–torsional nystagmus is commonly observed in superior vestibular neuritis. In this condition, dysfunction of the horizontal canal afferents contributes to the horizontal component, while dysfunction of the anterior canal afferents results in the torsional and slight upbeat components. However, it is important to note that a similar nystagmus pattern can also be seen in central disorders, such as lateral medullary syndrome (Wallenberg syndrome).

Isolated inferior vestibular neuritis causes torsional–downbeat nystagmus due to dysfunction in the posterior canal afferents. Downbeat nystagmus in IVN is torsional–downbeat, direction-fixed, suppressed by fixation, and beats toward the healthy ear. In contrast, central downbeat nystagmus is purely vertical, persists with fixation, often changes with gaze direction, and may be associated with cerebellar signs [29]. In their study from 2007, Wagner et al. [30] categorized “idiopathic DBN” into three distinct subgroups: (1) “pure” DBN (*n* = 17), presenting without additional neurological signs; (2) “cerebellar” DBN (*n* = 6), characterized by DBN with cerebellar signs but no evidence of cerebellar pathology on MRI; and (3) a “syndromic” form of DBN (*n* = 16), associated with at least two features such as bilateral vestibulopathy, cerebellar signs, or peripheral neuropathy [30]. It is crucial to identify the type of downbeating nystagmus to distinguish between the central and peripheral pathology, as the nystagmus in inferior vestibular neuritis can be easily considered a sign of central disorder.

The head impulse test (HIT) is the most important clinical modality for investigating the unilateral peripheral vestibular deficit. HIT is used to assess the vestibulo-ocular reflex (VOR) during rapid head movements. The presence of corrective catch-up saccades typically suggests peripheral vestibular hypofunction. Head impulse tests performed in bedside examination are positive only in some patients with vestibular neuritis [31,32]. Corrective saccades cannot always be noticed by simple observation, so vHIT is better for observing covert saccades and exploring vertical canals [33,34,35]. The gain of the VOR can be measured either on the horizontal plane or in the RALP (Right Anterior–Left Posterior) and LARP (Left Anterior–Right Posterior) planes, which correspond to the diagonal vestibular planes that assess the vertical semicircular canals [36].

The most important feature that enables vHIT to characterize inferior vestibular neuritis is the capacity to evaluate the function of the vertical canals. A study from 2017 using vHIT to assess canal function found that superior vestibular neuritis involving the anterior and lateral semicircular canals was diagnosed in 43% of cases. In contrast, isolated dysfunction of the posterior canal was observed in only 9.1% of patients [37]. In IVN, pathological cVEMP (saccular dysfunction) and an abnormal vHIT for the posterior semicircular canal are commonly associated, as both structures are innervated by the inferior vestibular nerve. However, cases exist where only one test is abnormal, depending on the extent of nerve involvement [17,36]. In a study of 38 patients with vestibular neuritis, Zhang et al. [37] found that 31 had superior vestibular neuritis and 7 had total neuritis. Using VNG and vHIT recordings, they demonstrated that the direction of nystagmus correlates with VOR gain in the respective semicircular canal, therefore allowing the identification of deficits in each semicircular canal.

Caloric testing is considered a diagnostic hallmark for the diagnosis of vestibular neuritis, but it evaluates only the function of the lateral canal at a very low-frequency range [38]. Caloric irrigation assesses angular VOR at a frequency range of about 0.003 Hz. Unilateral weakness (UW) at caloric testing is still the gold standard for unilateral vestibular loss. Discrepancies between the caloric testing and the vHIT results have been observed over time [39]. In a study published in 2022, the discordance rate was 20.8% in vestibular neuritis patients [40]. Other studies also identified discrepancies between the bithermal caloric test and horizontal v HIT test in their study [41,42]. The results of the attempts to correlate calorics and vHIT suggest the fact that these are complementary tests. Caloric testing in the inferior vestibular neuritis is expected to have normal results because the superior branch of the nerve is unaffected [41].

The clinical evaluation of otolitic organs is performed through the subjective horizontal and subjective vertical [43]. Subjective visual vertical (SVV) and subjective visual horizontal (SVH) are important tools for evaluating spatial disorientation in patients with peripheral or central vestibular injuries. SVV specifically assesses the utricular function, with the measured deviation angle indicating the functionality of the utricle [44].

VEMP testing can be used to assess the otolitic function in individuals with vestibular neuritis [45]. Vestibular-evoked myogenic potential findings provide valuable insights into acute vestibular neuritis. oVEMP primarily reflects utricular function, while cVEMP is more indicative of saccular function [46,47]. When both the superior and inferior divisions are affected, patients exhibit abnormal cervical and ocular VEMPs along with caloric areflexia on the affected side. In cases of superior vestibular neuritis, abnormal ocular VEMPs and caloric weakness are observed, whereas inferior vestibular neuritis results in reduced cervical VEMPs only.

In a study published in 2009, Murofushi et al. [48] demonstrated that when VEMP testing was conducted in patients with vestibular neuritis, approximately half showed abnormal VEMP results on the affected side, while the other half had normal responses. These findings suggest that vestibular neuritis can be categorized into two groups based on the combined results of caloric testing and VEMP testing: superior vestibular neuritis and total vestibular neuritis (involving both superior and inferior divisions). Additionally, the combined use of electrical and acoustic stimulation allowed for the differentiation between labyrinthine and retro-labyrinthine lesions [49].

The rotary chair (RC) has limited applicability in the diagnosis of vestibular neuritis because it evaluates the activity of both labyrinths simultaneously. The velocity step rotation test can be used to measure the VOR gain, which is decreased in the acute phase for the affected ear [50]. Individual RC parameters have limited accuracy. The highest predictive accuracy for identifying peripheral vestibular injury can be achieved by combining caloric and rotational chair testing. In a comparative study on 77 patients with a peripheral vestibular deficit and 80 normal subjects, Maes et al. [51] concluded that selecting 0.01, 0.05, and 0.1 Hz SHAT rotations is recommended as the optimal protocol for rotatory testing and combining rotatory testing with caloric and VEMP testing provides a more comprehensive assessment of the vestibular system. 

Imaging is not used routinely in vestibular neuritis, as it typically does not reveal diagnostic findings. While some studies have reported enhancement of the vestibular nerve signal on high-dose gadolinium MRI (magnetic resonance imaging), imaging remains of limited clinical diagnostic value [52,53,54]. However, radiologic evaluation should be conducted in patients with suspected central lesions to rule out stroke or other central pathologies [54]. Differential vestibular testing predicts short-term outcomes in acute unilateral vestibular failure, with greater vestibular involvement linked to longer hospital stays and more severe symptoms, emphasizing the need for comprehensive assessment [55].

### 4.2. Diagnostic Workup for Inferior Vestibular Neuritis

A clinician should systematically assess vestibular function to confirm IVN and rule out central pathology. See Table 2 and Table 3.

## 5. Conclusions

The intricate anatomy of the vestibular nerve highlights its differential vulnerability to inflammatory processes, with the superior division particularly susceptible due to its longer bony canal, narrower passage, and special anatomical features. Clinical presentations of vestibular neuritis vary according to the affected division, with superior vestibular neuritis being more common and diagnostically distinct from the rare inferior vestibular neuritis.

Diagnostic modalities such as vHIT have enhanced the identification of canal-specific deficits, particularly in vertical canals. Additional tests, including VEMP, offer insights into otolithic organ involvement. These diagnostic tests enable the physician with the possibility to distinguish between superior and inferior nerve involvement.

Despite advances, radiologic imaging has a limited role in the diagnosis of vestibular neuritis diagnosis, emphasizing the need for a multifaceted approach that integrates clinical examination with advanced vestibular testing. By combining different methods, clinicians can achieve precise differentiation between peripheral and central vestibular disorders, improving diagnostic accuracy and patient outcomes and avoiding expensive imaging evaluation. The studies collectively emphasize the need for a multimodal approach to diagnosing vestibular neuritis.

IVN remains an underdiagnosed condition due to its subtle clinical presentation and frequent misclassification as a central disorder. The most reliable diagnostic approach integrates a combination of vestibular tests. A reduced gain in the posterior semicircular canal on vHIT is a key finding, indicating selective dysfunction of the inferior vestibular nerve. Additionally, absent or reduced cervical vestibular evoked myogenic potential cVEMP responses confirm saccular involvement, while normal caloric responses help differentiate IVN from superior vestibular neuritis, in which caloric weakness is expected. Given these findings, a multimodal diagnostic approach is essential for distinguishing IVN from other vestibular pathologies, ensuring timely and accurate diagnosis. Future research should focus on standardizing these diagnostic criteria and refining prognostic indicators to improve clinical outcomes for affected patients.

The combination of different tools not only improves diagnostic accuracy but also helps clinicians distinguish between superior and inferior VN, a distinction that is vital for targeted management and prognosis. As our understanding of VN continues to evolve, integrating these diagnostic modalities will remain essential for providing comprehensive care to patients.

By synthesizing current diagnostic strategies, this review offers a practical framework for assessing inferior vestibular neuritis. The incorporation of complementary diagnostic tools, such as vHIT, VEMP, and caloric testing, improves diagnostic accuracy and informs effective management. Clinicians should integrate these insights into their practice to enhance outcomes for patients with vestibular dysfunction.

## Figures and Tables

**Table 1:** Studies focused on inferior vestibular neuritis; ↓ symbol means “reduced” or “decreased”.

Study	Year	Total Patients	IVN Cases	Key Findings	vHIT	cVEMP	oVEMP	Caloric Testing
Aw et al. [8]	2001	33	2	First report of isolated IVN, identified via posterior canal dysfunction	↓ Posterior canal gain	Not reported	Not reported	Normal
Halmagyi et al. [9]	2002	2	2	Selective inferior VN cases confirmed by VEMP and vHIT	↓ Posterior canal gain	Absent	Normal	Normal
Monstad et al. [10]	2006	3	3	Normal caloric results, loss of cVEMP responses	Not reported	Absent	Not reported	Normal
Zhang et al. [11]	2010	216	8	Normal caloric test, absent cVEMP, normal oVEMP	Not reported	Absent	Normal	Normal
Nola et al. [12]	2011	20	9	Differentiation between superior and inferior VN using VEMP	Not reported	Absent	Normal	Normal
Chihara et al. [13]	2012	71	13	Inferior VN group: normal calorics, abnormal cVEMP	Not reported	Absent	Normal	Normal
Shin et al. [14]	2012	41	3	Differentiation of VN subtypes using cVEMP and oVEMP	Not reported	Absent	Normal	Normal
Xie et al. [15]	2013	1	1	Case report: vHIT identified posterior canal dysfunction	↓ Posterior canal gain	Not reported	Not reported	Normal
Taylor et al. [16]	2016	43	1	Only 2.3% of VN cases were IVN, emphasizing its rarity	↓ Posterior canal gain	Not reported	Not reported	Normal
Büki et al. [17]	2017	44	4	IVN patients had misleading HINTS test results	↓ Posterior canal gain	Absent	Normal	Normal
Lee et al. [18]	2019	133	16	vHIT confirmed posterior canal deficit, normal caloric responses	↓ Posterior canal gain	Absent	Normal	Normal
Yacovino et al. [19]	2021	35	2	Comprehensive vestibular testing highlighted IVN diagnostic challenges	↓ Posterior canal gain	Absent	Normal	Normal

**Table 2:** Step 1: Clinical Examination (Bedside Tests).

Test	Expected IVN Result	Helps Differentiate From
Spontaneous Nystagmus	Torsional–downbeat, suppressed by fixation	Central pathology (persistent, gaze-evoked)
Head Impulse Test (HIT)	Normal in horizontal plane, abnormal in posterior canal	Superior VN (horizontal canal affected)
Subjective Visual Vertical (SVV)	Tilt toward affected side	Central disorders (random tilts)

**Table 3:** Step 2: Instrumental Testing for IVN, ↓ symbol means “reduced” or “decreased”.

Test	Expected IVN Result	Helps Differentiate From
Video Head Impulse Test (vHIT)	↓ Posterior canal gain with catch-up saccades	SVN (anterior/lateral canal affected)
Cervical VEMP (cVEMP)	Absent/reduced (saccular dysfunction)	Superior VN (normal cVEMP)
Ocular VEMP (oVEMP)	Normal (utricle intact)	Superior VN (oVEMP abnormal)
Caloric Testing	Normal (horizontal canal unaffected)	Superior VN (reduced response)

## Data Availability

All data are contained within the article.

## References

[1] Strupp M., Strupp M., Bisdorff A., Bisdorff A., Furman J., Furman J., Hornibrook J., Hornibrook J., Jahn K., Jahn K. (2022). Acute unilateral vestibulopathy/vestibular neuritis: Diagnostic criteria. J. Vestib. Res..

[2] Arbusow V., Schulz P., Strupp M., Dieterich M., Von Reinhardstoettner A., Rauch E., Brandt T. (1999). Distribution of herpes simplex virus type 1 in human geniculate and vestibular ganglia: Implications for vestibular neuritis. Ann. Neurol..

[3] Michael S., Thomas B., Marianne D. (2023). Vertigo and Dizziness, Common Complaints.

[4] Neuhauser H.K. (2007). Epidemiology of vertigo. Curr. Opin. Neurol..

[5] Sekitani T., Imate Y., Noguchi T., Inokuma T. (1993). Vestibular Neuronitis: Epidemiological Survey by Questionnaire in Japan. Acta Oto-Laryngol..

[6] Adamec I., Skorić M.K., Handžić J., Habek M. (2015). Incidence, seasonality and comorbidity in vestibular neuritis. Neurol. Sci..

[7] Hülse R., Biesdorf A., Hörmann K., Stuck B., Erhart M., Hülse M., Wenzel A. (2019). Peripheral Vestibular Disorders: An Epidemiologic Survey in 70 Million Individuals. Otol. Neurotol..

[8] Aw S.T., Fetter M., Cremer P.D., Karlberg M., Halmagyi G.M. (2001). Individual semicircular canal function in superior and inferior vestibular neuritis. Neurology.

[9] Halmagyi G.M., Aw S.T., Karlberg M., Curthoys I.S., Todd M.J. (2002). Inferior Vestibular Neuritis. Ann. N. Y. Acad. Sci..

[10] Monstad P., Økstad S., Mygland Å. (2006). Inferior vestibular neuritis: 3 cases with clinical features of acute vestibular neuritis, normal calorics but indications of saccular failure. BMC Neurol..

[11] Zhang D., Fan Z., Han Y., Yu G., Wang H. (2010). Inferior vestibular neuritis: A novel subtype of vestibular neuritis. J. Laryngol. Otol..

[12] Nola G., Guastini L., Crippa B., Deiana M., Mora R., Ralli G. (2011). Vestibular evoked myogenic potential in vestibular neuritis. Eur. Arch. Oto-Rhino-Laryngol..

[13] Chihara Y., Iwasaki S., Murofushi T., Yagi M., Inoue A., Fujimoto C., Egami N., Ushio M., Karino S., Sugasawa K. (2012). Clinical characteristics of inferior vestibular neuritis. Acta Oto-Laryngol..

[14] Shin B.-S., Oh S.-Y., Kim J.S., Kim T.-W., Seo M.-W., Lee H., Park Y.-A. (2012). Cervical and ocular vestibular-evoked myogenic potentials in acute vestibular neuritis. Clin. Neurophysiol..

[15] Xie S.J., Jia H.B., Xu P., Zheng Y.J. (2013). Inferior vestibular neuritis in a fighter pilot: A case report. Ear Nose Throat J..

[16] Taylor R.L., McGarvie L.A., Reid N., Young A.S., Halmagyi G.M., Welgampola M.S. (2016). Vestibular neuritis affects both superior and inferior vestibular nerves. Neurology.

[17] Büki B., Hanschek M., Jünger H. (2017). Vestibular neuritis: Involvement and long-term recovery of individual semicircular canals. Auris Nasus Larynx.

[18] Lee J.-Y., Park J.S., Kim M.-B. (2019). Clinical Characteristics of Acute Vestibular Neuritis According to Involvement Site. Otol. Neurotol..

[19] Yacovino D.A., Zanotti E., Cherchi M. (2021). The spectrum of acute vestibular neuropathy through modern vestibular testing: A descriptive analysis. Clin. Neurophysiol. Pr..

[20] Goebel J.A., O'Mara W., Gianoli G. (2001). Anatomic Considerations in Vestibular Neuritis. Otol. Neurotol..

[21] Gianoli G., Goebel J., Mowry S., Poomipannit P. (2005). Anatomic Differences in the Lateral Vestibular Nerve Channels and their Implications in Vestibular Neuritis. Otol. Neurotol..

[22] Büki B., Ward B.K. (2021). Length of the Narrow Bony Channels May Not be the Sole Cause of Differential Involvement of the Nerves in Vestibular Neuritis. Otol. Neurotol..

[23] Fetter M., Dichgans J. (1996). Vestibular neuritis spares the inferior division of the vestibular nerve. Brain.

[24] Kim J.-S., Kim H.J. (2012). Inferior vestibular neuritis. J. Neurol..

[25] Eggers S.D., Bisdorff A., von Brevern M., Zee D.S., Kim J.-S., Perez-Fernandez N., Welgampola M.S., Della Santina C.C., Newman-Toker D.E. (2019). Classification of vestibular signs and examination techniques: Nystagmus and nystagmus-like movements. J. Vestib. Res..

[26] Schwarz F.K., Vyskocil E., Büki B., Wiest G. (2022). Cupulolithiasis as an Alternative Mechanism for Pseudo-spontaneous Nystagmus in Horizontal Canal Benign Paroxysmal Positional Vertigo. OTO Open.

[27] Sun H., Wang Y., Jiang H., Gao Z., Wu H. (2022). The clinical application of head-shaking test combined with head-shaking tilt suppression test in distinguishing between peripheral and central vertigo at bedside vs. examination room. Braz. J. Otorhinolaryngol..

[28] Choi K.-D., Kim J.S., Kim H.-J., Koo J.-W., Kim J.H., Kim C.-Y., Oh C.W., Kee H.J. (2007). Hyperventilation-induced nystagmus in peripheral vestibulopathy and cerebellopontine angle tumor. Neurology.

[29] Cogan D.G. (1968). Down-Beat Nystagmus. Arch. Ophthalmol..

[30] Wagner J.N., Glaser M., Brandt T., Strupp M. (2007). Downbeat nystagmus: Aetiology and comorbidity in 117 patients. J. Neurol. Neurosurg. Psychiatry.

[31] Beynon G.J., Jani P., Baguley D.M. (1998). A clinical evaluation of head impulse testing. Clin. Otolaryngol..

[32] Hamid M. (2005). More than a 50% canal paresis is needed for the head impulse test to be positive. Otol. Neurotol..

[33] MacDougall H.G., Weber K.P., McGarvie L.A., Halmagyi G.M., Curthoys I.S. (2009). The video head impulse test. Neurology.

[34] Halmagyi G.M., Chen L., MacDougall H.G., Weber K.P., McGarvie L.A., Curthoys I.S. (2017). The Video Head Impulse Test. Front. Neurol..

[35] Jorns-Haderli M., Straumann D., Palla A. (2007). Accuracy of the bedside head impulse test in detecting vestibular hypofunction. J. Neurol. Neurosurg. Psychiatry.

[36] McGarvie L.A., Martinez-Lopez M., Burgess A.M., MacDougall H.G., Curthoys I.S. (2015). Horizontal Eye Position Affects Measured Vertical VOR Gain on the Video Head Impulse Test. Front. Neurol..

[37] Zhang X., Deng Q., Liu Y., Li S., Wen C., Liu Q., Huang X., Wang W., Chen T. (2023). Characteristics of spontaneous nystagmus and its correlation to video head impulse test findings in vestibular neuritis. Front. Neurosci..

[38] Molnár A., Jassoy B.D., Maihoub S., Mavrogeni P., Tamás L., Szirmai Á. (2023). Long-term follow-up of patients with vestibular neuritis by caloric testing and directional preponderance calculation. Eur. Arch. Oto-Rhino-Laryngol..

[39] van de Berg R., Rosengren S., Kingma H. (2018). Laboratory examinations for the vestibular system. Curr. Opin. Neurol..

[40] Waissbluth S., Sepúlveda V. (2022). Dissociation between Caloric and Video Head Impulse Tests in Dizziness Clinics. Audiol. Res..

[41] Lee J.-Y., Kwon E., Kim H.-J., Choi J.-Y., Oh H.J., Koo J.-W., Kim J.-S. (2020). Dissociated Results between Caloric and Video Head Impulse Tests in Dizziness: Prevalence, Pattern, Lesion Location, and Etiology. J. Clin. Neurol..

[42] Lee S.-U., Park S.-H., Kim H.-J., Koo J.-W., Kim J.-S. (2016). Normal Caloric Responses during Acute Phase of Vestibular Neuritis. J. Clin. Neurol..

[43] Zakaria M.N., Wahat N.H.A., Zainun Z., Sakeri N.S.M., Salim R. (2020). The Test-Retest Reliability of Subjective Visual Horizontal Testing: Comparisons between Solid and Dotted Line Images. J. Audiol. Otol..

[44] Zhang L., Ouyang S., Chen L., Huang H., Ou Y., Tang X. (2023). Evaluation of subjective visual vertical and horizontal in patients with acoustic neuroma based on virtual reality. Front. Neurosci..

[45] Murofushi T. (2016). Clinical application of vestibular evoked myogenic potential (VEMP). Auris Nasus Larynx.

[46] Murofushi T., Nakahara H., Yoshimura E., Tsuda Y. (2011). Association of air-conducted sound oVEMP findings with cVEMP and caloric test findings in patients with unilateral peripheral vestibular disorders. Acta Oto-Laryngol..

[47] Choi J.-Y. (2020). Vestibular-evoked myogenic potentials: Principle and clinical findings. Ann. Clin. Neurophysiol..

[48] Murofushi T. (2009). Detection of the lesion site in vestibular disorders using vestibular evoked myogenic potentials. Equilib. Res..

[49] Baloh R.W., Jacobson K.M., Beykirch K., Honrubia V. (1989). Horizontal Vestibulo-ocular Reflex after Acute Peripheral Lesions. Acta Oto-Laryngol..

[50] Ahmed M.F., Goebel J.A., Sinks B.C. (2009). Caloric Test Versus Rotational Sinusoidal Harmonic Acceleration and Step-Velocity Tests in Patients With and Without Suspected Peripheral Vestibulopathy. Otol. Neurotol..

[51] Maes L., Vinck B.M., Wuyts F., D'Haenens W., Bockstael A., Keppler H., Philips B., Swinnen F., Dhooge I. (2011). Clinical usefulness of the rotatory, caloric, and vestibular evoked myogenic potential test in unilateral peripheral vestibular pathologies. Int. J. Audiol..

[52] Karlberg M., Annertz M., Magnusson M. (2004). Acute Vestibular Neuritis Visualized by 3-T Magnetic Resonance Imaging With High-Dose Gadolinium. Arch. Otolaryngol. Neck Surg..

[53] Byun H., Chung J.H., Lee S.H., Park C.W., Park D.W., Kim T.Y. (2018). Clinical value of 4-hour delayed gadolinium-Enhanced 3D FLAIR MR Images in Acute Vestibular Neuritis. Laryngoscope.

[54] Wainberg F., Laffue A., Gualtieri F. (2022). Usefulness 3 tesla magnetic resonance imaging in the evaluation of central acute vestibular syndrome (P15-12.005). Neurology.

[55] Rambold H.A. (2015). Prediction of Short-Term Outcome in Acute Superior Vestibular Nerve Failure: Three-Dimensional Video-Head-Impulse Test and Caloric Irrigation. Int. J. Otolaryngol..

